# Are long nursing shifts on hospital wards associated with sickness absence? A longitudinal retrospective observational study

**DOI:** 10.1111/jonm.12643

**Published:** 2018-07-05

**Authors:** Chiara Dall’Ora, Jane Ball, Oliver Redfern, Alejandra Recio‐Saucedo, Antonello Maruotti, Paul Meredith, Peter Griffiths

**Affiliations:** ^1^ National Institute for Health Research Collaboration for Leadership in Applied Health Research and Care (Wessex) University of Southampton Southampton UK; ^2^ Faculty of Health Sciences University of Southampton Southampton UK; ^3^ School of Computing University of Portsmouth Southampton UK; ^4^ Dipartimento di Scienze Economiche Politiche e delle Lingue Moderne, Libera Università Maria Ss Assunta Roma Italy; ^5^ Research & Innovation Department Portsmouth Hospitals NHS Trust Portsmouth UK

**Keywords:** 12‐hour shifts, nursing, shift work schedule, sick leave

## Abstract

**Objective:**

To investigate whether working 12 hr shifts is associated with increased sickness absence among registered nurses and health care assistants.

**Background:**

Previous studies reported negative impacts on nurses’ 12 hr shifts; however, these studies used cross‐sectional techniques and subjective nurse‐reported data.

**Methods:**

A retrospective longitudinal study using routinely collected data across 32 general inpatient wards at an acute hospital in England. We used generalized linear mixed models to explore the association between shift patterns and the subsequent occurrence of short (<7 days) or long‐term (≥7 days) sickness absence.

**Results:**

We analysed 601,282 shifts and 8,090 distinct episodes of sickness absence. When more than 75% of shifts worked in the past 7 days were 12 hr in length, the odds of both a short‐term (adjusted odds ratio = 1.28; 95% confidence index: 1.18–1.39) and long‐term sickness episode (adjusted odds ratio = 1.22; 95% confidence index: 1.08–1.37) were increased compared with working none.

**Conclusion:**

Working long shifts on hospital wards is associated with a higher risk of sickness absence for registered nurses and health care assistants.

**Implications for Nursing Management:**

The higher sickness absence rates associated with long shifts could result in additional costs or loss of productivity for hospitals. The routine implementation of long shifts should be avoided.

## 1 BACKGROUND

Reducing sickness absence is high on health care employers’ agenda, in the context of staff shortages and the adverse effect on overall productivity and costs (Johnson, Croghan, & Crawford, 2003; Letvak & Buck, 2008; NHS Employers, 2014). Sickness absence is costly to the economy; figures from England's NHS put costs of staff absence due to poor health at £2.4 bn a year, accounting for around £1 in every £40 of the total budget (The Health Foundation, 2015). Furthermore, high rates of absenteeism among health care staff have been associated with lower patient satisfaction (Duclay, Hardouin, Sebille, Anthoine, & Moret, 2015).

Workplace factors have been shown to play a significant role in increasing sickness absence (Hahtela et al., 2015; National Institute for Health & Care Excellence, 2008). Among these factors, aspects of shift work, such as long shifts have been associated with decreases in employees’ well‐being and increases in sickness absence (Fekedulegn et al., 2013; Michie & Williams, 2003). A systematic review found limited evidence for the effect of aspects of shift work on sickness absence, concluding that evening work was associated with higher sickness absence levels (Merkus et al., 2012).

While the effects of shift work are typically job specific, there is an emerging body of evidence suggesting that long shifts in hospital wards may have adverse effects on staff, such as higher job dissatisfaction, burnout and intention to leave the job (Dall'Ora et al., 2015; Stimpfel & Aiken, 2013; Stimpfel, Brewer, & Kovner, 2015; Stimpfel, Sloane, & Aiken, 2012). Nonetheless, there is a trend toward the increasing use of long shifts for nursing staff on hospital wards (Merrifield, 2017a). These shifts are often adopted because of perceived efficiencies and to compensate for staff shortages (NHS Evidence, 2010).

There are recurring limitations of shift work research published to date: previous research has relied almost entirely on self‐report measures (Dall'Ora et al., 2016; Harma et al., 2015). However, self‐reported data do not correlate well with administrative records of sickness absence (Gaudine & Gregory, 2010; Grovle et al., 2012). Furthermore, a major limitation of previous shift work studies, being mostly cross‐sectional, has been the inability to meet a basic requirement for causal inference—demonstrating that cause precedes effect (Antonakis, Bendahan, Jacquart, & Lalive, 2010). Thus, it remains unclear whether there is any causal link between long shifts and objective sickness absence and the trend toward the increasing use of long shifts for nursing staff on hospital wards continues (Merrifield, 2017a).

Longitudinal studies that are able to capture the impact of different shift characteristics and to make use of formal records of sickness absence are needed. Therefore, the aim of this study was to examine the extent to which shift patterns are associated with sickness absence within a sample of nursing staff working on hospital wards.

## 2 METHODS

This was a retrospective longitudinal observational study using routinely collected data on nursing staff, shift data and sickness absence data. Within England's NHS hospitals, the nursing workforce is composed of registered nurses (RN) and variously titled health care assistants or health care support workers (HCA) who provide “hands on” care. The study took place in all inpatient general adult wards (32 wards) in a large acute care hospital trust in the South of England. The data examined for this study were derived from a larger parent study (ISRCTN registration: 17930973 http://www.isrctn.com/ISRCTN17930973). The University of Southampton Ethics Committee granted ethical approval to undertake this research (Submission Number 18,311).

### 2.1 Data extraction

Data on all shifts scheduled for RNs and HCAs over a 3‐year period (April 2012 to 31 March 2015) were extracted directly from a hospital‐wide electronic system (E‐Roster) that feeds into the payroll system. Data were also extracted for additional shifts worked in the same hospital by staff beyond their contracted hours. Both data sources were combined to achieve a record of all shifts worked on the study wards by all registered nurses and health care assistants employed in the hospital. Data were pseudo‐anonymized so shifts worked by the same individual could be linked, but no personal identifiable information were shared with the research team.

### 2.2 Outcome measures

Episodes of sickness absence were obtained from the appropriate records coded in the electronic rostering system. We also included absence recorded as “unauthorized” as these could include sickness periods when the proper reporting procedure was not followed, although the absolute number of such episodes was very low (*n* = 360, less than 1% of absences).

We considered that a sickness episode started on the first day the employee was absent from work and finished as soon as the employee went back to work for at least one shift. If the sickness absence involved 7 or more days of consecutive absence from work, including non‐working days, it was defined as long‐term sickness absence; if it was shorter than 7 consecutive days, it was classified as a short‐term sickness absence episode. This is in line with the UK government regulations, which requires employees to obtain a Statement of Fitness for Work from a general practitioner or hospital doctor if they are absent for 7 consecutive days or longer (UK Government, 2017) and with previous research that has adopted this threshold (Ferrie et al., 2005).

### 2.3 Shift work measures

We derived the following measures from the data set: shift length, type of shift (i.e., early, late, night, long day), and sickness absence episodes. Shift length was calculated as the difference between shift end and start time. Consequently, shift length was inclusive of breaks. Shifts were classified as “day” and “night” shifts based on the end time of the shift. If a shift finished before 8 a.m., it was classified as a night shift. For the purposes of descriptive and multivariable regression analysis we grouped shift length into three categories: 8 hr or less (≤8 hr), more than 8—less than 12 hr (>8–<12 hr), 12 hr or more (≥12 hr). In order to examine the effect of shift characteristics on sickness absence, a calculation of shift characteristics worked in the past 7 days was performed.

We calculated:

- The proportion of shifts worked in the past 7 days that were long (≥12 hr)
- The proportion of shifts worked in the past 7 days that were night shifts
- The proportion of days in the past 7 days that were worked
- Total number of hours worked in the past 7 days

Because the analysis was performed at the shift level, as opposed to the nurse level, no information was available on rotation status. However, by controlling for the proportion of night shifts worked in the past 7 days, shift rotation could be taken into account at least partially (i.e., those working 100% of their shifts as night shifts were likely to be those working on permanent night shifts schedules).

### 2.4 Statistical analysis

We performed descriptive analyses of sickness absence, including short‐term and long‐term absence episodes. We then examined sickness absence by nurses’ shift characteristics. The association between shift work characteristics and sickness absence was explored with generalized linear mixed models.

In order to assess the within‐ward and the within‐staff member variation for sickness, we computed intraclass correlation coefficients (ICC) from unconditional random intercept models. The ICC revealed that most of the variation in sickness episodes occurred at the individual nurse level (ICC = 0.32), with almost no variation occurring at the ward level (ICC = 0.004). This low ICC did not justify the inclusion of ward as a random effect in the models (Lee, 2000).

All analyses were performed at the shift level. To account for the fact that shifts were nested in staff members (i.e., to account for individual variation), nursing staff ID was included as random effects in the model. We also included staff role (registered nurse vs health care assistant) as levels of sickness are known to vary between staff groups (NHS Digital, 2017). To exclude multicollinearity we tested each model for the variance inflation factor (VIF); all VIF scores were <10, indicating low multicollinearity (Dormann et al., 2013).

We first explored the likelihood of a shift being missed based on its scheduled length. Secondly, we explored the likelihood of a sickness episode based on the proportion of long shifts worked over the past 7 days. We added the proportion of night shifts and the proportion of worked shifts over the past 7 days and staff role as control variables. We performed subgroup analyses of short‐term and long‐term sickness absence. All data preparation and analyses were undertaken using R (version 3.4.0) (R Studio Team., 2016), with mixed‐effects modelling using the lme4 package (version 1.1–13) (Bates, , Mächler, , Bolker, & Walker, 2015).

## 3 RESULTS

Our sample consisted of 601,282 shifts. The shifts were worked by 1944 staff members; of these, 1,244 were RNs and 700 were HCAs (including 88 staff members that worked shifts as both HCA and RN). There were 38,051 shifts lost due to sickness absence (6.3%) corresponding to 8,090 separate sickness episodes.

Overall, 1689 staff (86%) experienced at least one sickness episode during the 3‐year study period. The sickness episodes ranged from 1 day to 496 days in length; the most common length of sickness episodes was 2 days (*n* = 1,221, 15.1%). 2,532 (31.3%) sickness episodes lasted 7 or more days and were classified as long term sickness episodes, while the 5,555 sickness episodes lasting less than 7 days were classified as short term sickness episodes.

Forty‐eight percent of shifts worked lasted 8 hr or less (*n* = 270,709), and 38% of shifts were 12 hr or more (*n* = 216,877). The majority of day shifts lasted 8 hr or less (*n* = 270,390, 67.6%) and most of the night shifts lasted 12 hr or more (*n* = 110,022, 67.3%). The length of shift differed by staff group; RNs worked a higher number of long shifts than HCAs (40.7% vs. 33.7%). Distributions of shift work characteristics by shift length categories can be found in Table 1.

**Table 1:** Shift characteristics distribution by shift length category

	Shift length category, *n* (%)			
	≤8 hr	>8–<12 hr	≥12 hr	Total
Time of day				
Day	291,474 (66.6)	27,837 (6.3)	118,581 (27.1)	437,892 (100)
Night (shifts finishing at ≤8 a.m.)	319 (0.2)	53,053 (32.5)	110,018 (67.3)	163,390 (100)
Staff grade				
RNs	180,209 (46.7)	49,922 (13)	155,969 (40.3)	386,100 (100)
HCAs	111,584 (51.9)	30,968 (14.4)	72,630 (33.7)	215,182 (100)

If a shift was scheduled to be 12 hr or more in length, it was more likely to be missed due to sickness absence (OR = 1.24; 95% CI: 1.16–1.31), compared with a shift of 8 hr or less. Odds of both short‐term (OR = 1.18, 1.10–1.26) and‐long term (OR = 1.37, 1.23–1.53) sickness episodes were increased significantly when a shift was scheduled to last 12 hr or more. While shifts of between 8 and 12 hr were not associated with a significant increase in the overall odds of sickness, they were associated with an increase in long term sickness absence (OR 1.27; 95% CI: 1.11–1.46) (Table 2).

**Table 2:** Association of scheduled shift length and overall sickness absence, short‐term and long‐term sickness absence

	Overall sickness absence		Long term sickness absence (≥7 days)		Short term sickness absence (<7 days)	
Scheduled shift length	OR	95% CI	OR	95% CI	OR	95% CI
≤8 hr shift (reference category)						
>8–<12 hr	1.03	0.95–1.12	1.27*	1.11–1.46	0.92	0.83–1.01
≥12 hr	1.24*	1.16–1.31	1.37*	1.23–1.53	1.18*	1.10–1.26

Generalized linear mixed model; random effect: Staff ID.

*Statistically significant at *p* <.05.

The percentage of sickness episodes was lower when no long shifts were worked in the past 7 days (1.4%), compared with working more than three quarters of shifts as long shifts (1.7%). Working more than three quarters of shifts in the past 7 days as night shifts showed the highest percentage of sickness episodes (1.6%). The lowest proportion of days reflected a higher percentage of sickness episodes, and the highest proportions of days worked showed the lowest percentage of sickness episodes (Table 3).

**Table 3:** Shifts variables worked in the past 7 days and sickness absence

	Number of sickness episodes, *n* (%)	Number of non‐sickness shifts, *n* (%)	Total number of shifts, *n* (%)
Proportion of long shifts (≥12 hr) over worked shifts in past 7 days (%)			
0	3,855 (1.4)	280,820 (98.6)	284,675 (100)
>0–≤25	204 (1.2)	16,911 (98.8)	17,115 (100)
>25–≤50	744 (1.6)	46,109 (98.4)	46,853 (100)
>50–≤75	604 (1.4)	42,253 (98.6)	42,857 (100)
>75	2,683 (1.7)	155,110 (98.3)	157,793 (100)
Proportion of night shifts over worked shifts in past 7 days (%)			
0	6,183 (1.5)	413,951 (98.5)	420,134 (100)
>0–≤25	160 (1.1)	15,302 (98.9)	15,462 (100)
>25–≤50	432 (1.5)	27,950 (98.5)	28,382 (100)
>50–≤75	264 (1.4)	19,001 (98.6)	19,265 (100)
>75	1,051 (1.6)	64,999 (98.4)	66,050 (100)
Proportion of days worked in past 7 days (%)			
>0–≤25%	2,326 (1.9)	120,291 (98.1)	122,617 (100)
>25–≤50%	2,347 (1.6)	142,854 (98.4)	145,201 (100)
>50–≤75%	3,117 (1.2)	247,280 (98.8)	250,397 (100)
>75%	300 (1)	30,778 (99)	31,078 (100)

Working shifts of 12 hr or more in the past 7 days was associated with an increase in sickness absence, after adjusting for other shift variables. If more than 75% of shifts worked in the past 7 days were 12 hr or more in length, the odds of experiencing a sickness episode were increased by 27%, compared with working no 12 hr or more shifts (OR = 1.27; 95% CI: 1.18–1.37). Sickness absence was also significantly associated with the proportion of night shifts, days worked and grade of staff (Table 4).

**Table 4:** Associations of proportion of ≥12 hr shifts worked in past 7 days and sickness absence

Shift characteristics	AOR	95%CI
Proportion of ≥12 hr shifts over shifts worked in past 7 days (0% reference category)		
>0–≤25	1.12	0.97–1.29
>25–≤50	1.26*	1.15–1.37
>50–≤75	1.16*	1.05–1.28
>75	1.27*	1.18–1.37
Proportion of days worked over past 7 days (25% reference category)		
>25–≤50	0.91*	0.86–0.97
>50–≤75	0.77*	0.73–0.82
>75	0.66*	0.58–0.75
Proportion of night shifts over shifts worked in past 7 days (0% reference category)		
>0–≤25	0.91	0.78–1.07
>25–≤50	1.11	1.00–1.23
>50–≤75	1.06	0.94–1.21
>75	1.12*	1.03–1.21
Nurse grade		
Health care assistant (HCA) (Reference category)		
Registered nurse (RN)	0.65*	0.58–0.73

Generalized linear mixed model; random effect: Staff ID.

*Statistically significant at *p* <.05.

There was a high correlation between the proportion of days worked in the past 7 days and the number of total hours worked (correlation coefficient = 0.85). Because there was a high correlation between these variables, we dropped the total number of hours and controlled for proportion of worked shift only. A sensitivity analysis suggested this had no material effect on estimates of coefficients for other parameters.

Subgroup analyses of long‐term and short‐term sickness absence revealed similar effects to those observed in the general sample. The odds of experiencing a short‐term sickness absence episode were higher for all staff working >25% of their shifts as 12 hr or more, with higher odds for those working >75% of their shifts as 12 hr or more (AOR = 1.28, 95% CI: 1.18–1.44). For long‐term sickness absence, the only significant association was found for staff working >75% of their past shifts as ≥12 hr or more, compared with those working no 12 hr or more shifts at all (AOR = 1.22, 95% CI: 1.08–1.39), although all other proportions of long shifts were associated with non‐significant increases (Table 5).

**Table 5:** Association of proportion of 12 hr shifts and long/short‐term sickness absence

	Long‐term sickness absence (≥7 days)		Short‐term sickness absence (<7 days)	
Shift characteristics	AOR	95% CI	AOR	95% CI
Proportion of ≥ 12 hr shifts over shifts worked in past 7 days (0% reference category)				
>0–≤25	1.14	0.87–1.51	1.11	0.94–1.31
>25–≤50	1.12	0.95–1.32	1.30*	1.18–1.44
>50–≤75	1.18	0.99–1.41	1.14*	1.02–1.27
>75	1.22*	1.08–1.37	1.28*	1.18–1.39

Generalized linear mixed model; random effect: Staff ID.

*Statistically significant at *p* <.05.

Since night shifts in our sample were longer in length (67.3% of night shifts lasted 12 hr or more), we ran the models with interaction terms between long shifts and night shifts but the relationship was not significant, suggesting that the effect of shifts of 12 hr or more remains constant, regardless of whether shifts are worked in the day or night. It has been suggested that working long shifts is associated with a compressed working week, namely working longer but fewer days (Bambra, Whitehead, Sowden, Akers, & Petticrew, 2008); therefore, we explored the interaction between long shifts and worked days, but the relationship was not significant. The Pearson correlation coefficient between long shifts and worked days was.20, indicating a weak correlation.

## 4 DISCUSSION

To our knowledge, this is the first study to use longitudinal data and objective shift and outcome measures to explore the association between long shifts and sickness absence in registered nurses and health care assistants on hospital wards. We found that staff scheduled to work a shift of 12 hr or more were 24% more likely to miss the shift due to sickness absence, compared with staff who were scheduled to work shifts of 8 hr or less.

While occasional shifts of 12 hr or more (<25%) in the past 7 days were not significantly associated with more sickness absence, when staff worked a higher proportion of shifts of 12 hr or more, sickness rates increased, with the highest odds for those working more than three quarters of their shifts as 12 hr or more shifts. Higher proportions of long shifts were associated with both long and short‐term sickness absence, although for long‐term sickness the only significant association was observed when staff had worked more than three quarters of their past shifts as long shifts.

These findings are consistent with previous work showing that working long shifts are associated with a higher likelihood of reporting adverse staff outcomes, including burnout and job dissatisfaction (Ball et al., 2017; Dall’Ora et al., 2015; Stimpfel et al., 2012). These factors may in turn lead to sickness absence (Schaufeli, Bakker, & Van Rhenen, 2009). Our study shows that shifts of 12 hr or more are associated with both short‐term and long‐term sickness absence. Long‐term sickness is likely to reflect a health impairment process (Bakker, Demerouti, de Boer, & Schaufeli, 2003) and the longer‐term health consequences of working long shifts need to be further explored. Long shifts have been associated with higher levels of fatigue (Barker & Nussbaum, 2011; Chen, Davis, Daraiseh, Pan, & Davis, 2014). Fatigue is a well‐established predictor of sickness absence, suggesting that it may play a mediating role between long shifts and sickness absence (Janssen, Kant, Swaen, Janssen, & Schröer, 2003; Sagherian et al., 2017).

A previous study found that when nurses were working 8 hr shifts, they were more likely to report missed shifts than those working 12 hr shifts (Stone et al., 2006). However, if nurses worked 12 hr shifts as part of a compressed week, they worked fewer shifts overall, and so there were fewer shifts to be missed. Our study found that even when adjusting for days worked, the negative effect of higher proportions of long shifts remained significant, indicating that having a higher number of days off might not mitigate the negative effects of long shifts. These findings mirror evidence that the choice of the compressed work week for shift workers may be in conflict with the recommended criteria of a safe shift system, and health may be compromised (Kecklund, Eriksen, & Akerstedt, 2008).

In England, the USA and several European Union countries, shifts of 12 hr or more are becoming the norm and appear to be popular with some nurses, who are often reported preferring long shifts because of the greater number of days off work, compared with nurses working 8 hr or less shift patterns (Ball, Dall’Ora, & Griffiths, 2015). Long shifts have been introduced as a strategy to reduce staffing costs by reducing the overlaps between shifts (NHS Evidence, 2010), with some NHS Trusts currently implementing mandatory 12.5 hr shifts for nursing staff hospital‐wide (Merrifield, 2017a). However, if long shifts are associated with higher rates of sickness, any benefits could be undermined. Increased sickness absence may lead to the increased use of agency staff to fill the vacant shift; this represents a costly option that the NHS is increasingly trying to avoid (NHS Improvement, ). Furthermore, agency nurses called to fill a vacant shift may be less productive and less effective (Bae, Mark, & Fried, 2010).

## 5 LIMITATIONS

This study had some limitations. Firstly, this was a single site study, and these findings may not generalize to other hospitals. However, a previous study has noted that there tends to be more variation in shift patterns within hospitals than there is between hospitals in England (Griffiths et al., 2014). Furthermore, we were not able to account for staff characteristics including age and personal commitments external to work, both of which may influence responses to shift work. However, the inability to control for staff characteristics, including age and chronic illness, could lead to bias if such staff characteristics were systematically associated with staff electing to work specific shift patterns. Unless age and chronic illness were associated with an increased preference for longer rather than shorter shifts, which seems unlikely from the limited available anecdotal evidence (Ball et al., 2015), the resulting bias would lead to underestimating the adverse effects of longer shifts. Furthermore, since staff ID was included as a random effect in our models (i.e., shifts were nested in individual staff members), individual characteristics were at least partially controlled for.

The ability to rest within and between shifts is an important factor in relation to shift work (Tucker, Smith, Macdonald, & Folkard, 1999; Wendsche, Ghadiri, Bengsch, & Wegge, 2017). We could not track shifts worked for other employers and whether staff were able to take scheduled breaks within shifts. If these factors are related to shift length they may partially explain the associations we observed, although they do not detract from the significance of the findings. Lastly, we were unable to determine the cause of sickness which could illuminate the mechanism through which long shifts might generate increased sickness. However, the association of long shifts with long‐term sickness and absence does indicate that long shifts are associated with potentially significant health problems. Future research should include cost‐effectiveness analyses on the effects of higher proportion of ≥12 hr shifts and further shift variables that are likely to have an impact on sickness absence.

## 6 CONCLUSIONS

When nurses and health care assistants work high proportions of shifts of 12 hr or more, sickness absence is increased. As well as indicating worse health for employees, such increases may undermine a key motivation for introducing the 12 hr shift pattern—organisational efficiency. Further moves toward routine implementation of long shifts should be reconsidered and neither organisational benefits nor benefits for staff should be assumed.

## 7 IMPLICATIONS FOR NURSING MANAGEMENT

Nurse managers are often required to organise, approve and review nursing staff members’ schedules. The main goal for nurse managers is to ensure that the nursing workforce is configured so that safe quality of patient care can be delivered. As this research has found, how aspects of shift work are organised has implications for nurses’ sickness absence. The findings of this study indicate that working high proportions of shifts of 12 hr or more is likely to increase sickness absence in registered nurses and health care assistants. This finding, despite being far from the description of an “ideal” shift system in nursing, may offer further knowledge to nurse managers aiming to maintain and improve their employees’ job attendance and well‐being.

If staff were able to take breaks during their shifts, if the workload during shifts were not exceedingly high and if there were enough staff on the wards to complete care activities, the effect of 12 hr shifts may be different. However, health care systems are struggling with an increasing nursing shortage (Attree et al., 2011), high rates of vacancies filled with temporary and agency staff (NHS Improvement, 2016) and anecdotal reports of lack of breaks during the shifts due to understaffing (Merrifield, 2017b). In this current context, these findings suggest that, while occasional 12 hr shift work may not have adverse consequences, working higher proportions may lead to higher sickness absence. Therefore, nurse managers should question the routine implementation of long shift patterns, especially if this is based on assumed cost savings.

## ETHICAL APPROVAL

An ethics application was submitted to the University of Southampton's ethics committee through ERGO and was granted approval by the Research Governance Office (Submission Number 18,311).
